# Supplementary material for: From sequence to enzyme mechanism using multi-label machine learning
Source: BMC Bioinformatics. 2014 May 19;15:150. doi: 10.1186/1471-2105-15-150 (PMC4229970; doi:10.1186/1471-2105-15-150)
Supplement: Additional file 2 — Java code of ml2db. Additional file ml2db_code.tar.gz contains the Java source code to run the multi-label machine learning experiments and save the results to database. The code’s Javadoc is included. [file 1471-2105-15-150-S2.zip › additional file 2/ml2db/ml2db/doc/main/ml2db/graphviz/package-use.html]

Uses of Package main.ml2db.graphviz


JavaScript is disabled on your browser.


- Overview
- Package
- Class
- Use
- Tree
- Deprecated
- Index
- Help

- Prev
- Next

- Frames
- No Frames

- All Classes

# Uses of Package main.ml2db.graphviz

- Packages that use main.ml2db.graphviz

  | Package | Description |
  | --- | --- |
  |  |  |
  | --- | --- |
  | main.ml2db.graphviz |  |
  | test.ml2db.graphviz |  |
- Classes in main.ml2db.graphviz used by main.ml2db.graphviz

  Class and Description

  GraphViz

  Purpose: GraphViz Java API
  Description:
  With this Java class you can simply call dot from your Java programs
  Example usage:

  GraphVizArff

  Generates a GraphViz representation (.dot file) of the instances, class
  labels and attributes in a Weka/Mulan dataset and their relations.
- Classes in main.ml2db.graphviz used by test.ml2db.graphviz

  Class and Description

  GraphViz

  Purpose: GraphViz Java API
  Description:
  With this Java class you can simply call dot from your Java programs
  Example usage:

  GraphVizArff

  Generates a GraphViz representation (.dot file) of the instances, class
  labels and attributes in a Weka/Mulan dataset and their relations.

  GraphVizArffColoured

  Nodes and edges can of a base dot file can be coloured to show their labelled
  status (train set), or their correctly/uncorrectly predicted status (test
  set).

- Overview
- Package
- Class
- Use
- Tree
- Deprecated
- Index
- Help

- Prev
- Next

- Frames
- No Frames

- All Classes
